# Overexpression of GRB2 Enhances Epithelial to Mesenchymal Transition of A549 Cells by Upregulating SNAIL Expression

**DOI:** 10.3390/cells7080097

**Published:** 2018-08-07

**Authors:** Payal Mitra, Pazhanichamy Kalailingam, Hui Bing Tan, Thirumaran Thanabalu

**Affiliations:** 1 Department of Molecular Medicine, STRF, University of Texas Health San Antonio, 8403 Floyd Curl Dr, San Antonio, TX 78229, USA; mitrap@uthscsa.edu; 2 School of Biological Sciences, Nanyang Technological University, Singapore 637551, Singapore; kpazhanichamy@ntu.edu.sg (P.K.); hbing194@gmail.com (H.B.T.); 3 National University Health System (NUHS), 119228 Singapore, Singapore

**Keywords:** E-cadherin, cell adhesion, migration, actin cytoskeleton

## Abstract

GRB2 is an adaptor protein which interacts with phosphorylated TGF-β receptor and is critical for mammary tumour growth. We found that TGF-β1-induced EMT increased GRB2 expression in A549 cells (non-small cell lung cancer). Overexpression of GRB2 (A549^GRB2^) enhanced cell invasion while knocking down GRB2 (A549^GRB2KD^) reduced cell migration and invasion, probably due to increased vinculin and reduced Paxillin patches in A549^GRB2KD^ cell. TGF-β1-induced EMT was more pronounced in A549^GRB2^ cells and attenuated in A549^GRB2KD^ cells. This could be due to the reduced expression of E-cadherin in A549^GRB2^ and increased expression of E-cadherin in A549^GRB2KD^ cells, even before TGF-β1 stimulation. Expression of SNAIL was elevated in A549^GRB2^ cells and was further enhanced by TGF-β1 stimulation, suggesting that GRB2 down-regulates E-cadherin by enhancing the expression of SNAIL. The N-SH3 domain of GRB2 was critical for suppressing E-cadherin expression, while the C-SH3 domain of GRB2 mediating interaction with proteins such as N-WASP was critical for promoting invasion, and the SH2 domain was critical for suppressing E-cadherin expression and invasion. Thus, our data suggests that GRB2 enhances EMT by suppressing E-cadherin expression and promoting invasion probably through N-WASP to promote metastasis.

## 1. Introduction

Metastasis is responsible for most of cancer-related deaths [1]. Metastasis is a complex multistep process in which tumour cells migrate from the primary tumour site through the extracellular matrix, enter the blood circulation by forming new blood vessels (tumour angiogenesis) and migrate to distant organs and tissues (extravasation) and proliferate to form secondary tumours [2]. Epithelial cancer cells in primary tumours are strongly associated with each other via different types of cellular junctions such as tight junctions, adherens junctions and desmosomes [3]. Thus, tumour cells have to break these tight cellular junctions before invading the surrounding tissues as single cells [1]. Epithelial-to-Mesenchymal Transition (EMT) facilitates invasion by attenuating cell–cell adhesion, reorganising the actin cytoskeleton and up-regulating the expression of mesenchymal markers [4,5,6]. A variety of environmental stimuli such as extracellular matrix (collagen), and growth factors and cytokines such as transforming growth factor-β (TGF-β), Epidermal Growth Factor (EGF), Hepatocyte Growth Factor (HGF), and tumour necrosis factor-α (TNF-α) have been shown to induce EMT [7]. The transforming growth factor-β (TGF-β) family regulates many cellular processes, including cell differentiation, apoptosis, proliferation, migration, stem cell maintenance, regulation of immune system and others [8]. The three isoforms of TGF-β, TGF-β1, TGF-β2 and TGF-β3 are expressed ubiquitously and have been studied extensively for their role in cell physiology [8].

Binding of active TGF-β to the extracellular ligand binding domain of both the TGF-β receptors (TβRI and TβRII) causes phosphorylation and activation of TβRI by TβRII [9]. Activation of TβRI induces the phosphorylation of SMAD transcription factors that regulate a variety of physiological processes, such as EMT and chromatin remodelling [10,11]. In addition to its signalling through SMADS, TGF-β can also activate other signalling pathways, such as Ras/MAPK, PI3K and RHO-like GTPases mediated pathways, which also contribute to EMT [12,13,14,15,16]. Lung cancer is still one of the leading causes of cancer deaths, as it is often diagnosed at late stages of cancer with ineffective chemotherapies [17]. Various receptor tyrosine kinases such as Epidermal Growth Factor Receptor (EGFR), HGFR/c-Met along with their downstream signalling molecules, such as GRB2, mitogen-activated protein kinase (MAPK), and Phosphoinositide 3-kinase (PI3K), have been found to be over-expressed in lung cancer [17]. However, the role of GRB2 in receptor tyrosine kinase- or Ser/Thr kinase-activated signalling such as TGF-β in NSCLC or SCLC has thus far still not been clearly elucidated.

GRB2 is an ubiquitously expressed 25 kDa adaptor protein participating in many cellular functions, such as cell proliferation and cell survival, and is also a crucial regulatory link in various oncogenic signalling pathways [2]. GRB2 consists of one SH2 domain flanked by two SH3 domains [2]. The SH2 domain of GRB2 specifically binds to phosphorylated tyrosine residues of several growth factor-activated receptor tyrosine kinases, which include EGFR and c-Met (HGF receptor), among others. Both the SH3 domains have been found to interact with the guanine nucleotide exchange factor, Sos (Son of Sevenless) [18], which has been postulated to be constitutively associated with GRB2 in the cytoplasm [18]. Upon receptor activation, GRB2/Sos complex activates Ras and stimulates downstream Ras-MAPK pathway [2].

GRB2 localises primarily in the cytoplasm and has also been found to localise to membrane ruffles, structures formed during epithelial cell migration, by immunofluorescence assay [19]. The localisation of GRB2 to the ruffles suggests that it might interact with proteins participating in the regulation of actin cytoskeleton. One such protein is Neural-Wiskott Aldrich Syndrome Protein (N-WASP), belonging to the WASP family of actin nucleation promoting factors [20]. These multi-domain WASP family proteins have been shown to activate the Arp2/3 complex, which is responsible for branched actin polymerisation, required for the formation of actin-rich structures such as lamellipodia and invadopodia, which play critical roles in cell migration and invasion [21]. Binding of GRB2 through its C-SH3 domain to N-WASP has been shown to activate N-WASP, which can stimulate actin nucleation by the Arp2/3 complex [21].

The role of GRB2 in tumour progression has been studied widely in breast tumour, as GRB2 has been found to be highly expressed in many types of cancers, especially breast tumours [2]. Galliher et al., 2007 demonstrated that Src kinase phosphorylated TβRII at Tyr284 upon stimulation with TGF-β, required for the activation of p38-MAPK (mitogen-activated protein kinase) through the intermediate binding of SH2 domain containing proteins such as GRB2 and Shc to TβRII in mammary epithelial cells [22]. GRB2/TβRII complex was also found to be required for the activation of p38MAPK in NMuMG (normal mammary epithelial) cells as knocking down the expression of GRB2 or expression of TβRII mutant, which cannot bind to GRB2 (Y284F-TβRII), significantly impaired the stimulation of p38-MAPK and the ability of TGF-β to induce EMT in NMuMG cells [23]. TβRI can also phosphorylate ShcA, upon TGF-β stimulation, which can then associate with GRB2/Sos complex to activate MAPK signalling pathway [24].

We found that the expression of GRB2 was up-regulated in A549 cells stimulated with TGF-β1, suggesting a role for GRB2 during TGF-β1-induced EMT. Overexpression of GRB2 in A549 cells (A549^GRB2^) enhanced TGF-β1-induced EMT with pronounced reduction of E-cadherin expression in A549^GRB2^ cells. More importantly, the expression of E-cadherin was downregulated, while that of SNAIL was upregulated, even before TGF-β1 stimulation, suggesting that GRB2 regulates E-cadherin expression independent of TGF-β1 signalling by upregulating SNAIL expression. A549^GRB2^ cells were highly invasive, while A549^GRB2KD^ cells exhibited reduced migratory and invasive characteristics, suggesting that GRB2 promotes EMT and invasion in A549 lung cancer cells. Expression of GRB2^P49L^ (N-terminal SH3 domain mutant) did not reduce E-cadherin expression but still promoted invasion; expression of GRB2^R86K^ (SH2 domain mutant) did not reduce E-cadherin expression and had reduced invasive characteristics; expression of GRB2^P206L^ (C-terminal SH3 domain mutant) reduced E-cadherin expression but attenuated invasion. Thus, our data suggest that the N-terminal SH3 is critical for promoting EMT while the C-terminal SH3 is critical for invasion and the SH2 domain which binds to phosphor-tyrosine is critical for both.

## 2. Materials and Methods

### 2.1. Cell Culture and Transfection

Human lung adenocarcinoma cell line A549 and Human Embryonic Kidney cells (HEK293T) (ATCC, Manassas, VA, USA) were maintained in growth media, GM (DMEM + 10% FBS and 100 IU/mL penicillin and 100 IU/mL streptomycin) at 37 °C in a 5% CO_2_ humidified atmosphere. GRB2 knockdown and overexpression studies were carried out using 3rd-generation lentiviral infections. EMT was induced by serum starving the cells for 12 h followed by incubation of the cells in serum Free DMEM with 5 ng/mL of TGF-β1 (Peprotech, Rocky Hill, NJ, USA) for 48 h.

### 2.2. DNA Constructs

GRB2 shRNA (cagatattcctgcgggacataCTCGAGtatgtcccgcaggaatatctgTTTTTG) was cloned in lentiviral plasmid pLJM1 (a gift from David Sabatini (Addgene plasmid # 19319) [25] under the transcriptional regulation of U6 promoter and used for knocking down expression of GRB2. Human His-tagged GRB2 gene, GRB2-His, was cloned in lentiviral plasmid pLJM1 and used for overexpression studies. GRB2 point mutant constructs were made by mutating the human GRB2 cDNA at specific amino acid residues at its N-SH3, SH2 and C-SH3 domains. The constructs were GRB2^P49L^-His (N-SH3 mutant), GRB2^R86K^-His (SH2 mutant), and GRB2^P206L^-His (C-SH3 mutant).

### 2.3. Lentivirus Preparation and Infection

HEK293T cells in a 10 cm dish at 80–90% confluency were transfected using polyethylene imine (PEI) with lentiviral plasmid (pLJM1) (empty vector, plasmid expressing human GRB2 specific shRNA or GRB2-His gene, (WT or GRB2 mutants) together with packaging plasmids VSVG (3.75 µg), REV (3.125 µg), pDNL (6.125 µg) and PLJM1 plasmid (18.75 µg). Twenty-four hours after the transfection, the media (containing the virus) was filtered through a 0.45 µm filter and used to infect A549 cells at 40% confluency and a second infection 24 h later.

### 2.4. Cell lysis and Western Blot Analysis

Cells were trypsinised and lysed in appropriate volume of RIPA lysis buffer (50 mM Tris–HCl, pH 7.5, 200 mM NaCl, 1%Triton X-100, 0.1% SDS, 0.5% sodium deoxycholate, 10% glycerol, 1 mM EDTA and 1 mM PMSF. The lysate was boiled in SDS-PAGE sample buffer at 100 °C for 5 min and 30 µg of proteins were resolved by 10% SDS-PAGE gel and transferred onto nitrocellulose membrane. The membrane was probed with the appropriate primary antibodies (anti-E-cadherin, anti-Vimentin and anti-N-cadherin, BD Biosciences, San Jose, CA, USA), (anti-GRB2 and anti-SNAI1, Santa Cruz Biotechnology, Santa Cruz, CA, USA) and developed using secondary antibody conjugated with horseradish peroxidase (Sigma-Aldrich, St. Louis, MO, USA). Anti-GAPDH (Ambion, Austin, TX, USA) was used as the loading control. Densitometric analysis was carried out using Image J software from three independent sets of experiments.

### 2.5. Immunofluorescence

A549 cells grown on coverslips were fixed, permeabilised, blocked and probed with appropriate primary antibodies followed by Alexa488-conjugated secondary antibody, while actin cytoskeleton was visualised using Alexa568-conjugated (red) phalloidin (Molecular Probes). Nucleus was visualised using DAPI staining. Fluorescence images were acquired using Olympus IX51 fitted with Cool SNAP^HQ^ camera and analysed using Metamorph software (Molecular Devices).

### 2.6. Migration Assay

A549 cells, 3 × 10^5^ per well were seeded in a 6-well plate and grown to confluency. A scratch was made using a sterile micropipette tip and a time lapse was set up to observe the cell migration using the Olympus microscope IX81 fitted with Photometrics camera over a period of 33 h. In each experiment, at least 12 randomly selected regions were monitored. Size of the scratch was measured using Metamorph software. The minimal separation between the opposing cell fronts was measured.

### 2.7. Matrigel Invasion Assay

Transwell inserts (Costar) of 8.0 µm pore size were coated with Matrigel diluted at 1:10 with serum-free media and allowed to gel overnight at 37 °C in an incubator with 5% CO_2_. The next day, A549 cells were trypsinized and 2.5 × 10^5^ cells in 200 µL serum-free media were added to the upper chamber of the Matrigel-coated invasion inserts. The well below was filled with 600 µL of complete media and the cells were incubated in the cell culture incubator for around 40 h. Cells from the upper chamber were removed and the cells that had invaded through the Matrigel were fixed with 3.7% formaldehyde for 2 min, washed twice with 1X PBS and stained with 1% crystal violet in methanol for 15 min at room temperature, and washed, and the cells were visualised under a 10× objective lens with an inverted microscope.

### 2.8. Soft Agar Assay

Culture dishes were coated with 1.0% noble agar (Sigma) in complete media (1.5 mL agar/well in a 6-well plate) and allowed to solidify at room temperature for 20 min. Meanwhile, cells were trypsinized, counted (25,000 cells/mL) and mixed with 0.6% noble agar and added to agar-coated wells. The top layer with the cells was allowed to solidify for another 20 min. 500 µL of complete media was added to each well to prevent drying, and they were incubated at 37 °C for a period of 15 days. Cells were then stained with 0.05% crystal violet for 30 min, washed with PBS and images of the colonies formed were captured using a 4× objective lens. The average number of colonies was counted manually, and the average colony area was measured using Image J software.

### 2.9. Experimental In Vivo Metastasis Assay

All the animal experiments carried out were approved by Institutional Animal Care and Use Committee (IACUC) of Nanyang Technological University (NTU). Nude mice (CrTac:NCr-Foxn1nu, InVivos Pte Ltd. (Singapore)) were injected with 200 μL (5 × 10^5^ cells in total) of A549^Vect^ or A549^GRB2^ cells in the lateral tail vein (*n* = 5) and maintained in NTU animal house. The mice were sacrificed after 8 weeks by CO_2_ asphyxiation. Lung tissues were embedded in OTC, 5 µm cryosections on superfrost slides (Fisher), were stained with Haematoxylin and Eosin, and were mounted in DPX. Slides were imaged using 20× and 40× objectives.

### 2.10. Statistical Analysis

All the statistical data was generated from at least three independent experiments. Statistical analysis was performed using two-tailed unpaired student’s *t*-test to compare the difference between groups. Significance is indicated by stars * *p* < 0.05, ** *p* < 0.01, *** *p* < 0.001.

## 3. Results

### 3.1. Expression of GRB2 Was Elevated during TGF-β1-Induced EMT in A549 Cells

Expression of GRB2 has been reported to be elevated in human breast cancer biopsies [26], and the role of GRB2 in tumour progression has been studied widely in breast tumour [2]. The role of GRB2 in lung cancer has not been well characterised; thus, the expression and localisation of GRB2 during TGF-β1-induced EMT in A549 cells was investigated. A549 cells were seeded at 2 × 10^5^ cells/60 mm dish, grown to 25% confluency, serum-starved for 12 h, and stimulated with 5 ng/mL of TGF-β1 or left untreated. Cells were visualised for changes in their morphology followed by immunoblotting with anti-GRB2, anti-E-cadherin (epithelial marker), anti-N-cadherin (mesenchymal marker) and anti-GAPDH (loading control).

**Figure 1:**
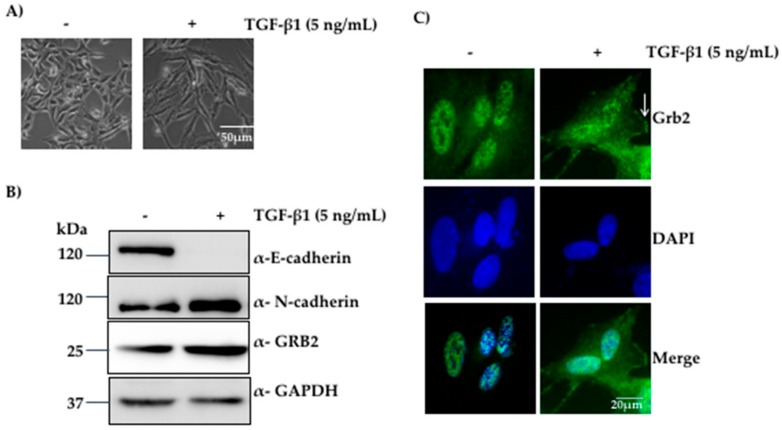
Expression of GRB2 was elevated during TGF-β1-induced EMT in A549 cells. (**A**) A549 cells were visualised under 10× objective after 48 h of incubation with or without 5 ng/mL of TGF-β1 at 37 °C; (**B**) Total cell lysate of untreated A549 cells and TGF-β1-treated cells were probed by anti-GRB2, anti-E-cadherin (epithelial marker), anti-N-cadherin (mesenchymal marker) and anti-GAPDH (loading control) antibodies; (**C**) A549 cells, untreated or TGF-β-stimulated, were immunostained using anti-GRB2 (green) and DAPI (blue).

We observed morphological changes after 24–27 h of TGF-β1 stimulation, the epithelial A549 cells started losing their cell–cell contacts, became elongated and adopted more mesenchymal and spindle-shaped phenotype. At the end of 48 h, most of the A549 cells displayed mesenchymal phenotype (Figure 1A). Western blot analysis of the protein extracts from these cells showed a reduction in the expression of E-cadherin and an increase in the expression of N-cadherin compared to the control, suggesting that EMT had taken place. We also found that the expression of GRB2 increased in TGF-β1 treated cells compared to the control (Figure 1B), and this was not due to increased transcription, as determined by qPCR (Figure S1), suggesting that GRB2 is stabilised by TGF-β1 treatment. The results suggest that GRB2 may play a positive role in signalling pathways mediated by TGF-β1 in A549 cells.

GRB2 is known to localise in the cytoplasm in most of the cell types [19]. However, it has also been reported to localise both in the cytoplasm and the nucleus in both normal and tumour breast tissue [26]. Thus, we characterised the localisation of GRB2 after TGF-β1-induced EMT. A549 cells were seeded on coverslips, grown to 40% confluency and EMT was induced as described above. After 48 h of TGF-β1 treatment, the cells were fixed, permeabilized, probed with anti-GRB2 and secondary antibody conjugated with Alexa Fluor 488. Nuclei were visualised using DAPI stain. In control untreated cells, GRB2 could not be detected in cytoplasm; upon TGF-β1 stimulation, GRB2 was found in the cytoplasm, especially close to the plasma membrane, where it may be participating in TGF-β1-stimulated signalling pathways. This suggests that TGF-β1 stimulation localised GRB2 to the plasma membrane, where it interacts with proteins of the signalling pathway.

### 3.2. Overexpression of GRB2 Enhanced TGF-β1-Induced EMT in A549 Cells

**Figure 2:**
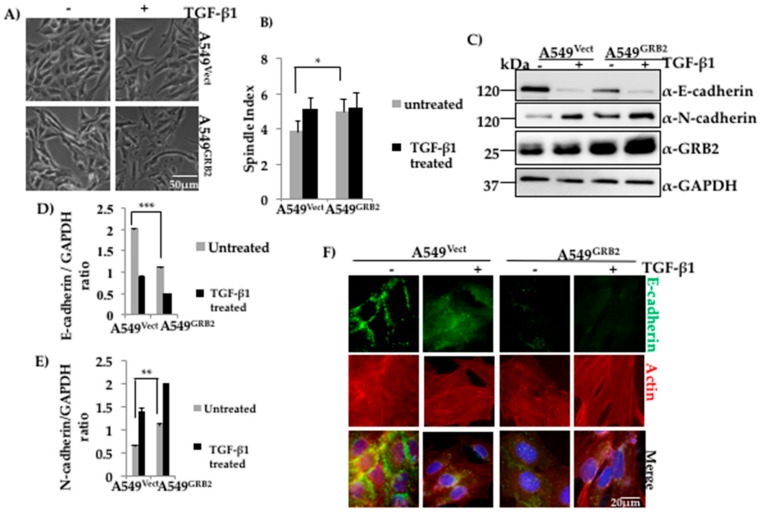
Overexpression of GRB2 enhanced TGF-β1-induced EMT in A549 cells. (**A**) Control (A549^Vect^) and GRB2 overexpressing (A549^GRB2^) A549 cells were grown in a 60 mm dish and induced to undergo EMT as described in Figure 1. After 48 h of EMT induction, images were captured using a 10× objective lens to study morphological changes after TGF-β1 stimulation; (**B**) Spindle Index, which is the ratio of the maximum length to the maximum width of the cell, was calculated for A549^Vect^ and A549^GRB2^ cells before and after TGF-β1 stimulation using Image J software. Experiments were carried out in duplicate to calculate significance; (**C**) A549^Vect^ and A549^GRB2^ cells were then scraped, lysed, and the protein extracts were subjected to Western blot analysis using antibodies against E-cadherin, N-cadherin and GRB2. GAPDH was used as the loading control; (**D**) Densitometric analysis of E-cadherin expression level from panel C was carried out using Image J software. (*** *p* < 0.001); (**E**) Densitometric analysis of N-cadherin expression level from panel C was carried out using Image J software. (*** *p* < 0.001); (**F**) A549^Vect^ and A549^GRB2^ were seeded on coverslips and EMT was induced with TGF-β1 as described in Figure 1. After 48 h of induction of EMT, the cells were fixed and immunostained using antibodies against E-cadherin (green) and actin (red). DAPI was used to stain the nucleus. Images were taken under a 40× oil immersion lens.

Expression of GRB2 was found to be enhanced in A549 cells upon TGF-β1 stimulation, suggesting a possible role for GRB2 in TGF-β1-induced EMT (Figure 1B). In order to study the role of GRB2 overexpression during TGF-β1-induced EMT, as well as other cellular processes that occur during EMT, we generated A549^GRB2^ stable cells using 3rd-generation lentivirus, which expressed GRB2-His. The plasmid (pLJM-GRB2-His) or empty vector, together with packaging plasmids, were transfected in HEK293T cells and the viral supernatant was used to infect A549 cells. The infection efficiency was ~90% (data not shown) and cells were selected with puromycin (2 µg/mL) to remove the uninfected cells. Induction of EMT of A549^GRB2^ cells with TGF-β1 stimulation caused more pronounced separation and elongation compared to EMT induction of A549^Vect^ cells (Figure 2A). On calculating the spindle index, the ratio of maximum length to maximum width of a cell, a significant increase (23%) was shown in the spindle index of A549^GRB2^ cells compared to the A549^Vect^ cells before TGF-β1 stimulation (Figure 2B), indicating that overexpression of GRB2 increased the spindle phenotype of A549 cells. The expression of GRB2 was verified using Western blot analysis (Figure 2C). The stable cells were induced to undergo EMT for 48 h at 37 °C as described above, lysed, and the expression levels of E-cadherin and N-cadherin were determined by Western blot. We found that the E-cadherin expression level was significantly lower (~50%) in A549^GRB2^ cells compared to the A549^Vect^ cells before EMT induction with TGF-β1 (Figure 2C,D) and the N-cadherin expression level was higher (~30%) in A549^GRB2^ cells compared to the A549^Vect^ cells after TGF-β1 stimulation (Figure 2C,E). This observation suggests that GRB2 overexpression may not only enhance TGF-β1-induced EMT, but also induce an EMT-like phenotype, even before stimulation with TGF-β1 in A549 cells. E-cadherin is responsible for maintaining the epithelial cell morphology, cell–cell adhesion and quiescence [27]. Therefore, we determined whether overexpression of GRB2 altered the localisation of E-cadherin at the cell–cell junction as well. To this end, immunostaining of A549^Vect^ and A549^GRB2^ before and after TGF-β1 stimulation was performed using anti-E-cadherin antibody. The localisation of E-cadherin at the cell–cell junction in A549^GRB2^ cells was reduced compared to A549^Vect^ cells even before the addition of TGF-β1 (Figure 2F). The cell morphology of untreated A549^GRB2^ cells was slightly elongated compared to the A549^Vect^ cells. This observation suggests that the reduction of E-cadherin expression results in the decrease of E-cadherin localisation at the cell–cell junction in A549 cells after TGF-β stimulation. We also carried out EMT in A549 cells with GRB2 knocked down, A549^GRB2KD^. TGF-β1 stimulation caused both the cells (A549Vect and A549^GRB2KD^) to undergo EMT in a similar manner (Figure S2A,B). Both A549^Vect^ and A549^GRB2KD^ cells were found to undergo loss of E-cadherin expression during EMT. However, the expression level of the epithelial marker, E-cadherin, was found to be higher in untreated A549^GRB2KD^ cells compared to the control (Figure S2C,D). However, expression of N-cadherin was found to be reduced inA549^GRB2KD^-treated cells (Figure S2C,E) compared to the contro,l indicating that GRB2 knockdown may cause partial EMT in TGF-β-induced A549 cells. Furthermore, no significant difference in the localisation of E-cadherin at the cell–cell junction was observed between A549^Vect^ and A549^GRB2KD^ cells after TGF-β stimulation (Figure S2F).

### 3.3. GRB2 Promotes Cell Migration and Reduced Vinculin Localisation in A549 Cells

**Figure 3:**
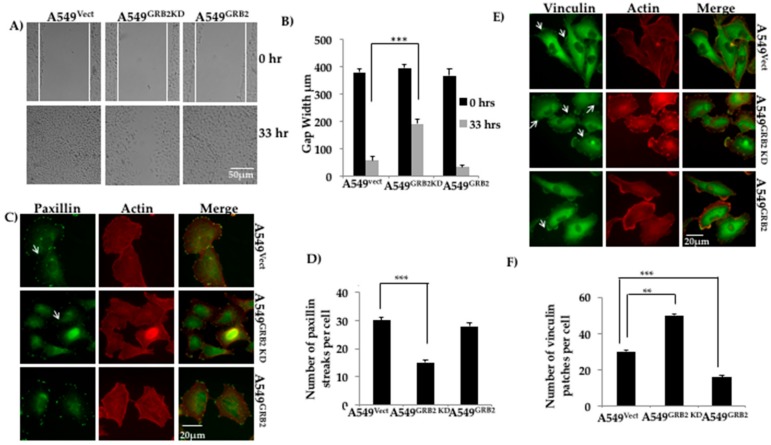
GRB2 promotes cell migration and reduced vinculin localisation in A549 cells. (**A**) A549^Vect^, A549^GRB2KD^ and A549^GRB2^ cells were seeded on a 6-well plate and grown to 100% confluency. A scratch was made on the monolayer and the cells were set for time lapse imaging in a chamber at 37 °C with 5% CO_2_ for a period of 48 h with live cell images being taken at 30 min intervals under a 10X objective lens. The time point at which the gap of the control cells was taken as the reference time point (33 h). Images from the 0 h and 33 h time points were considered for calculations; (**B**) Gap width was measured using Image J software. A total of 6 random fields per time point per sample were measured for each experiment. Three independent experiments were performed to calculate the significance value. (*** *p* < 0.001); (**C**) A549^Vect^, A549^GRB2KD^ and A549^GRB2^ cells were seeded on silane coated coverslips, grown to 40% confluency, fixed and immunostained using anti-Paxillin and Alexa488 secondary antibodies. Alexa568-Phalloidin was used to visualise F-actin. Images were observed under 40× oil immersion lens; (**D**) The number of Paxillin patches per cell was calculated manually. A total of 25 cells per cell were considered for an experiment. (*** *p* < 0.001); (**E**) A549^Vect^, A549^GRB2KD^ and A549^GRB2^ cells were seeded on silane-coated coverslips, grown to 40% confluency, fixed and immunostained using anti-Vinculin and Alexa488 secondary antibodies. Alexa568-Phalloidin was used to visualise F-actin. The images were taken under a 40× oil immersion lens; (**F**) The number of vinculin patches per cell was calculated manually. 25 cells per sample were counted for one experiment. Three independent experiments were carried out to calculate significance (** *p* < 0.01, *** *p* < 0.001).

Epithelial cancer cells acquire enhanced migratory capabilities to metastasise to secondary sites of tumour growth [4]. Proteins interacting with the actin cytoskeleton have been found to be up-regulated during the migration of tumour cells during EMT [4,28]. GRB2 has been shown to interact with proteins belonging to the WASP family of actin cytoskeleton regulators, such as WASP and N-WASP, at their proline-rich domains [2]. To study the role of GRB2 during A549 cell migration, a wound healing (scratch) assay was performed using A549^Vect^, A549^GRB2 KD^ and A549^GRB2^ cells. We found that the A549^GRB2^ cells migrated faster and closed the gap (<33 h) before the control A549^Vect^ cells, whereas the A549^GRB2KD^ cells migrated significantly slower than the control A549^Vect^ cells (Figure 3A,B). Thus, the results suggest that overexpression of GRB2 promotes cell migration and the increased expression of GRB2 during EMT may promote metastasis.

Paxillin localisation at the focal adhesion is a good indicator of increased migration in many highly metastatic cell lines [28]. To study the effect of GRB2 knockdown and overexpression on Paxillin distribution at focal adhesions in A549 cells, we seeded the A549^Vect^, A549^GRB2KD^ and A549^GRB2^ cells on silane-coated coverslips, fixed and immunostained for Paxillin. We observed that the number of Paxillin streaks per cell was significantly reduced in A549^GRB2KD^ cells compared to the control (Figure 3C,D). There was no significant difference in the localisation of Paxillin in A549^GRB2^ compared to A549^Vect^ cells (Figure 3C,D) and in the expression of Paxillin in A549 ^GRB2^ compared to A549^Vect^ cells (data not shown). The observation suggests that focal adhesion turnover in A549^GRB2KD^ cells could be affected, and this could be one of the reasons for the slower migration rate observed on GRB2-knockdown in A549 cells.

Vinculin is an actin-binding protein which localises to focal adhesion and regulates cell adhesion and migration [29]. Thus, we characterised the role of GRB2 in focal adhesion in A549 cells by immunostaining. A549^Vect^, A549^GRB2 KD^ and A549^GRB2^ cells were seeded on silane-coated coverslips, grown to 40% confluency, permeabilized and probed with anti-vinculin antibody, followed by secondary antibody conjugated with Alexa Fluor 488. The number of vinculin patches per cell was manually quantified. Vinculin patches in A549 cells appear as small dot-like structures at the cell periphery (Figure 3E), unlike in fibroblasts, where they localise as larger streaks or patches [30]. We found that the number of vinculin patches per cell was significantly reduced in A549^GRB2^ cells compared to the control cells, while the number of vinculin patches per cell increased in A549^GRB2KD^ cells compared to the control (Figure 3F). The observed differences in vinculin patches per cell were not due to changes in the expression level of vinculin (data not shown). Thus, our results suggest that GRB2 impedes the localisation of vinculin to focal adhesions in A549 cells.

### 3.4. N-Terminal SH3 Domain and Central SH2 Domain of GRB2 Are Critical for Suppressing E-Cadherin Expression

**Figure 4:**
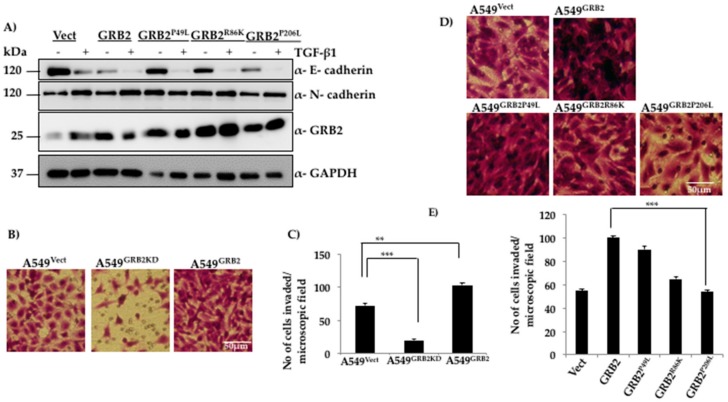
Functional N-terminal and C-terminal SH3 domains are critical for reducing E-cadherin expression and promoting invasion of A549 cells, respectively. (**A**) A549 cells were infected with lentivirus particles generated using vector, or plasmids expressing WT GRB2 or its mutants (GRB2^P49L^, GRB2^R86K^ and GRB2^P206L^), selected with puromycin, and EMT was as described in Figure 1. Cells were then lysed and the protein extracts were subjected to Western blot analysis by probing with antibodies against E-cadherin, N-cadherin, and GRB2. GAPDH was used as the loading control; (**B**) Matrigel-coated invasion assay inserts were seeded with 2 × 10^5^ cells of A549^Vect^, A549^GRB2KD^ and A549^GRB2^ in 200 µL of serum-free media on the upper chamber of the insert. The well below was filled with complete media containing 20% FBS. The cells were incubated for 40 h at 37 °C, the cells which had invaded through the Matrigel were fixed, washed and stained with crystal violet, and imaged under a 10× objective lens; (**C**) Cells in four random fields per sample were counted to quantify the number of cells that had invaded. Three independent sets of experiments were carried out to calculate significance. (** *p* < 0.01, *** *p* < 0.001); (**D**) Matrigel-coated invasion assay inserts were seeded with 2 × 10^5^ cells of A549^Vect^, A549^GRB2^, A549^GRB2P49L^, A549^GRB2R86K^ and A549^GRB2P206L^ per 200 µL of serum-free media in the upper chamber of the insert. The well below was filled with complete media containing 20% FBS. The cells were incubated for 40 h at 37 °C; the cells that had invaded through the Matrigel were fixed, washed and stained with crystal violet and imaged under a 10× objective lens; (**E**) Four random fields per sample were imaged and the cells were counted to quantify the number of cells that had invaded through the Matrigel. Three independent sets of experiments were carried out to calculate significance. (** *p* < 0.01, *** *p* < 0.001).

GRB2 is an adaptor protein with a SH3-SH2-SH3 domain organisation and overexpression of GRB2 enhances TGF-β1 EMT induction (Figure 2) and reduces E-cadherin expression even before EMT induction (Figure 2C). In order to characterise the role of individual domains of GRB2 during TGF-β1-induced EMT, the following site-directed mutations were used to disrupt the function of each domain: P49L (N-SH3 mutant), R86K (SH2 mutant) and P206L (C-SH3 mutant). The plasmids expressing these mutants were used to generate lentivirus and infect A549 cells to generate stable cell lines. The stable cell lines (A549^Vect^, A549^GRB2^, A549^GRB2P49L^, A549^GRB2R86K^, A549^GRB2P206L^) were induced to undergo EMT as described above. A549 cells expressing three mutants were found to undergo EMT upon TGF-β1 stimulation, as determined by the reduction in E-cadherin expression in A549 cells expressing any of the three mutants. However, expression of GRB2^P49L^ or GRB2^R86K^ mutants in A549 did not reduce E-cadherin expression before TGF-β1 treatment, as observed in A549^GRB2^ cells (Figure 4A). This observation suggests that the N-SH3 and SH2 domains of GRB2 are critical for repression of E-cadherin expression in A549 cells.

### 3.5. C-Terminal SH3 Domain and Central SH2 Domain of GRB2 Is Critical for Promoting Invasion of A549 Cells

The ability of cancer cells to break the ECM barrier, invade the surrounding tissues and enter the blood circulation allows them to metastasise to distant organs and tissues. Since GRB2 expression was found to increase during EMT in A549 cells, the effect of GRB2 on the invasive properties of A549 cells was studied. A Matrigel invasion assay was carried out with A549^Vect^, A549^GRB2KD^ and A549^GRB2^ cells. We found that a significantly lower number of A549^GRB2KD^ cells invaded through the Matrigel compared to the A549^Vect^ cells upon staining with crystal violet, suggesting that knocking down GRB2 expression impedes A549 cells from invading the ECM barrier (Figure 4B,C). On the other hand, the invasive ability of A549^GRB2^ cells was significantly higher compared to the control, suggesting that GRB2 enhances the invasive ability of A549 cells.

In order to characterise the domain of GRB2 necessary for enhancing invasion in A549 cells, a Matrigel invasion assay was carried out with A549^Vect^, A549^GRB2^, A549^GRB2P49L^ A549^GRB2R86K^ and A549^GRB2P206L^ cells. We found that the number of cells that had invaded through the Matrigel was significantly greater in A549 cells expressing either GRB2 or GRB2^P49L^ mutant compared to the control (A549^Vect^), and there was no significant difference in the invasive capabilities of A549^GRB2^ and A549^GRB2P49L^ cells (Figure 4D,E), suggesting that N-SH3 domain-mediated interactions are not critical for cell invasion. A549^GRB2R86K^ cells showed a significant reduction in the number of cells invading through the Matrigel compared to A549^GRB2^ cells. However, the most significant reduction in the invasive capacity of A549 cells was observed with the C-SH3 point mutant, GRB2^P206L^, when compared to wild type GRB2. The C-terminal SH3 domain is known to interact with proline-rich proteins such as N-WASP, which has been known to be required for invasion [31]. This suggests that the C-terminal SH3 domain, along with the SH2 domain, plays a critical role in regulating the invasive capacity of A549 cells mediated by GRB2.

### 3.6. The Expression of SNAIL Is Increased in GRB2-Overexpressing A549 Cells

**Figure 5:**
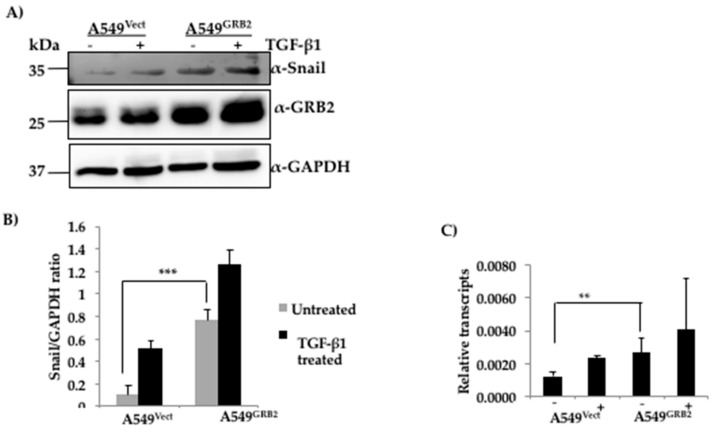
Expression of SNAIL is increased in GRB2 overexpressing A549 cells. (**A**) Protein extracts from A549^Vect^ and A549^GRB2^ cells, before and after TGF-β1 treatment, were subjected to Western blot using antibodies against Snail and GRB2. GAPDH was used as the loading control; (**B**) Densitometric analysis of SNAIL expression level was carried out using Image J software. (*** *p* < 0.001); (**C**) RNA was extracted from the samples using Trizol reagent, converted to cDNA and used to carryout qPCR using Snail primers. MRPL27 was used as the control. Relative abundance of GRB2 normalised against MRPL27 for two independent experiments was plotted (** *p* < 0.01, *** *p* < 0.001).

The changes in the gene expression profiles during the induction of EMT are regulated by master transcription factors such as SNAIL, TWIST and ZEB [4]. It is widely accepted that SNAIL binds to the promoter of E-cadherin and represses E-cadherin expression in many epidermal cell types undergoing EMT [32]. Expression of SNAIL is also known to induce EMT and fibroblastic phenotype in many epithelial cell lines [32]. However, the involvement of GRB2 in the regulation of E-cadherin has not been characterised to date. We found that overexpression of GRB2 caused a reduction in the expression of E-cadherin even before TGF-β1 stimulation. Hence, we wanted to study whether the regulators of E-cadherin expression such as SNAIL or SLUG were up-regulated in GRB2 overexpressing A549 cells. Protein lysates from A549^Vect^ and A549^GRB2^ cells, before and after TGF-β1-induced EMT, were collected and subjected to Western blot analysis using antibodies against SNAIL and SLUG. SLUG expression could not be detected (data not shown). SNAIL, being an inducer of EMT, was found to be induced in the TGF-β1 treated control cells but not in the untreated control cells (Figure 5A). Interestingly, we detected a significant expression of SNAIL protein in GRB2 overexpressing cells even before TGF-β1 stimulation and the level increased slightly after TGF-β1 stimulation in GRB2- overexpressing cells. In order to determine if the increased SNAIL protein expression was due to increase in transcription we carried out RT-PCR analysis. Total RNA was isolated from A549^Vect^ and A549^GRB2^ overexpressing cells, before and after TGF-β1 stimulation, reverse transcribed to cDNA and used to carry out qPCR to quantify SNAIL mRNA levels. We found that there was a significant increase in the SNAIL mRNA levels in A549^GRB2^ cells before TGF-β1 stimulation compared to the control (Figure 5C). This suggests that increased expression of transcription factor SNAIL by GRB2 overexpression could be one of the possible mechanisms for the reduction in E-cadherin expression in A549^GRB2^ cells. ERK2 phosphorylation has been shown to enhance EMT [33], and we found that overexpression of GRB2 enhanced ERK phosphorylation and this was further enhanced by TGF-β1 treatment (Figure S3), suggesting that GRB2 may enhance EMT by stabilising phospho-ERK2 (Figure S3).

### 3.7. Overexpression of GRB2 Enhanced Anchorage-Independent Growth of A549 Cells

**Figure 6:**
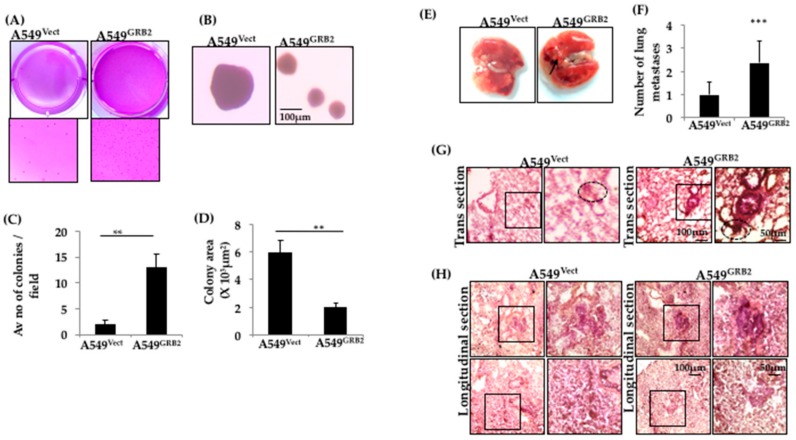
Overexpression of GRB2 enhanced anchorage-independent growth of A549 cells and in vivo lung metastasis. (**A**) A549^Vect^ and A549^GRB2^ cells were subjected to a soft agar colony formation assay, where A549 cells were allowed to form colonies for 15 days, stained with 0.05% crystal violet, and imaged. Digital images of the wells are shown; (**B**) Images of the colonies were captured using a 4× objective lens of a phase contrast microscope and used for counting the number colonies and measuring area of colonies; (**C**) Colonies in ten random fields per sample were counted to quantify the average number of colonies/microscopic field. Three independent sets of experiments were carried out to calculate significance; (**D**) Ten colonies per sample were used to quantify the average colony area using Image J software. Three independent sets of experiments were carried out to calculate significance; (**E**) Digital images of the lungs from mice (*n* = 5) injected with A549^Vect^ and A549^GRB2^ cells, respectively, were taken. Representative images are shown here; (**F**) The number of lung metastases in individual mice were counted under a microscope. 5 mice per sample were considered to calculate significance (*** *p* < 0.001); (**G**) Images of lung sections were taken from lung cryosections of the mice injected with A549^Vect^ and A549^GRB2^ cells using a 4× objective lens of a phase contrast microscope. Lung metastases are circled. Representative images are shown here; (**H**) Images of lung metastases for the longitudinal and transverse cryosections of mice injected with A549^Vect^ and A549^GRB2^ cells were taken using a 4× objective lens. Metastases are highlighted for each sample. Representative images are shown here.

The ability of cancer cells to exhibit anchorage-independent growth, the colony-forming ability in semi-solid media, has been used as a marker to identify the metastatic potential of many cancer cell types in vitro [34]. The role of GRB2 in anchorage-independent growth of A549 lung cancer cells was studied by carrying out a soft agar colony formation assay. Equal numbers of A549^Vect^ and A549^GRB2^ cells were added to soft agar and allowed to form colonies for a period of 2 weeks, after which they were stained with crystal violet and counted using a 4X objective lens. We found that A549^Vect^ cells formed around 2 colonies per microscopic field, with an average colony area of around 6 × 10^3^ μm^2^. A549^GRB2^ cells formed significantly higher numbers of colonies (13) per microscopic field; however, the average colony area was reduced significantly to 2 × 10^3^ μm^2^ (Figure 6C,D). The decrease in the colony area in A549^GRB2^ cells could potentially be due to the decrease in the growth rate caused by GRB2 under anchorage-independent conditions [35], or it could be due to reduced E-cadherin expression in A549^GRB2^ cells. This result suggests that GRB2 regulates the anchorage-independent growth of A549 cells. We verified that this effect of GRB2 on the colony-forming ability of A549 cells was not related to the proliferation rate of the cells in anchorage-dependent normal growth media conditions (data not shown).

### 3.8. Overexpression of GRB2 Enhances In Vivo Lung Metastasis

The ability of GRB2 to enhance TGF-β1-induced EMT, expression of mesenchymal transcription factor SNAIL, migration, invasion and anchorage-independent growth in A549 lung cancer cells led us to further investigate the role of GRB2 in in vivo metastatic progression.

An experimental metastasis assay was used to study late-stage metastatic progression in immunocompromised mice by injecting the human cancer cells directly into its systemic circulation to observe the development of distant metastases. A549^Vect^ and A549^GRB2^ cells were injected into the lateral tail vein of nude mice, and cancer metastasis to different organs, primarily pulmonary metastasis, was monitored for a period of 8 weeks. Morphologically, secondary growth on the surface of the lungs was more prominent in all the mice (*n* = 5) injected with A549^GRB2^ cells compared to A549^Vect^ cell-injected mice (Figure 6E). Upon staining the longitudinal and transverse lung tissue sections with H&E, we found that the histology of lungs from A549^Vect^-injected mice was visually different from the lungs of A549^GRB2^-injected mice. One of the main structures found in the lung tissue are the alveoli, characterised by a single epithelial layer of cells. Alveoli in the A549^GRB2^-injected mice were more uniform, with continuous epithelial layer staining. However, alveoli in the A549^Vect^-injected mice were not even, with epithelial layer staining all over the alveolar sacs (Figure 6G). Interestingly, the area around the bronchioles, characterised by thick epithelial layer staining (as shown in the magnified image of the A549^GRB2^ panel in Figure 6G) was darkly stained, indicating a cluster of cells, possibly a secondary growth or tumour. However, such darkly stained clusters of cells were found to be fewer in number and visually lower in intensity in A549^Vect^-injected mice. The longitudinal tissue sections of both groups also showed these abnormal masses of cells (Figure 6H). These darkly stained masses of cells were considered to be individual metastases and were counted under a microscope. Quantification of the number of metastases showed significant increase in the number of metastases in mice injected with A549^GRB2^ cells compared to A549^Vect^ (Figure 6F). All these results together indicated that overexpression of GRB2 enhanced in vivo lung metastases.

## 4. Discussion

EMT is an orchestrated series of molecular, genetic and cellular changes that transforms polarised epithelial cells to mesenchymal cells with increased cell migration and invasive phenotype through the loss of cell polarity and loss of cell–cell junctions [36]. TGF-β is a potent inducer of EMT in many cell types [37]. GRB2 has been found to be overexpressed in many tumours, such as breast cancer, along with EGF receptor and components of the RAS/MAPK signalling pathway [26]. Studies on breast cancers have revealed that formation of GRB2: TβRII complex is essential for mammary cancer growth mediated by TGF-β [23]. However not much is known about the role of GRB2 in EMT during lung cancer progression.

Expression of GRB2 was found to increase upon TGF-β1 stimulation of A549 cells (Figure 1B), but this is probably due to the enhanced stability of GRB2 or translation as TGF-B1 stimulation did not enhance transcription of GRB2 (Figure S1). The localisation of GRB2 was altered after TGF-β1 stimulation in A549 cells, and GRB2 was found in both the nucleus and cytoplasm (Figure 1C), suggesting an active role for GRB2 during EMT. GRB2 has previously been reported to localise in the nucleus in breast tumours [26]. Though no specific role for GRB2 in the nucleus has been characterised so far, it could regulate the expression of genes involved in EMT, as overexpression of GRB2 in A549 cells enhanced EMT compared to the control (Figure 2), with a significant decrease in E-cadherin expression even before TGF-β1 stimulation. This observation supports our hypothesis that GRB2 promotes EMT in A549 cells. Localisation of E-cadherin at cell–cell junction was also found to be visibly reduced in GRB2 overexpressing cells before TGF-β1 stimulation (Figure 2D). Knocking down the expression of GRB2 did not affect the reduction of E-cadherin expression during EMT, but there was no increased expression of N-cadherin; however, E-cadherin expression in A549^GRB2KD^ cells was higher than in the control A549^Vect^ cells (Figure S2). The accompanying increase in N-cadherin expression also did not occur in A549^GRB2KD^ cells, suggesting that the partial changes due to reduction in the expression of GRB2 may be compensated by other proteins.

Cell migration and invasion are important cellular processes during tumour metastasis, and epithelial cells display increased motility during EMT, which helps them to invade the vascular system and metastasise to distant organs [5]. Both these properties were found to be regulated by GRB2 in A549 cells, as knocking down GRB2 expression reduced cell migration and invasion, while overexpression of GRB2 enhanced cell invasion without enhancing cell migration. We also found that the number of vinculin patches per cell were reduced in A549^GRB2^ cells and increased in the A549^GRB2KD^ cells compared to the control (Figure 3E,F). We have previously found that reduced motility correlates with increased number of vinculin patches [30]. Expression of Paxillin and its phosphorylated form has been found to be a good indicator of cell motility in highly metastatic cell lines [38]. Furthermore, we found that the localisation of Paxillin at the focal adhesions in A549^GRB2KD^ cells decreased significantly compared to the control cells (Figure 3C,D). A549^GRB2KD^ cells, which migrated slower, had reduced Paxillin patches, increased vinculin patches and increased E-cadherin expression, suggesting that cell migration is significantly reduced in the GRB2 knockdown cells, probably due to increased cell–cell adhesion as a consequence of increased E-cadherin expression and changes in the composition of focal adhesion proteins. GRB2, being an adaptor protein, is crucial in regulating various cellular processes by interacting with a wide variety of proteins [2]. Hence, its necessity in A549 cell migration and invasion processes may be due to its involvement in the regulation of multiple signalling pathways, as well as its ability to regulate actin cytoskeleton by activating N-WASP [21].

Anchorage-independent growth capability of cancer cells has been linked to their metastatic potential and used as a method to connect in vitro and in vivo cancer phenotypes [34]. The drastic increase in the colony numbers of A549 cells upon GRB2 overexpression indicated that GRB2 plays a crucial role in regulating the anchorage-independent growth of A549 cells. The reduced size of the colony could be due to reduced expression and localisation of E-cadherin in A549^GRB2^ cells (Figure 2F). This observation further strengthens our hypothesis that GRB2 probably promotes cancer progression by regulating the later stages of metastasis.

In order to identify the domains responsible for the reduced E-cadherin expression, as well as the increased invasion mediated by GRB2, we analysed the effect of overexpressing GRB2 mutants (GRB2^P49L^, GRB2^R86K^, GRB2^P206L^) in A549 cells. Data from the expression of the GRB2 mutants suggests that the C-terminal SH3 domain of GRB2 is critical for invasion, the N-terminal SH3 domain of GRB2 is critical for suppression of E-cadherin expression, while the SH2 domain of GRB2 is critical for both invasion and reduction of E-cadherin expression. The N-terminal SH3 domain of GRB2 has been shown to bind to Sos (GEF for Ras) with a very high affinity, the SH2 domain is known to interact with phosphorylated tyrosine residues of many growth factor receptors [2], while the C-terminal SH3 domain has been shown to bind to and activate N-WASP, which regulates the actin cytoskeleton [21]. As the SH2 domain binds to phosphorylated tyrosine residues on growth factors, it may play a role in targeting GRB2 and GRB2-bound proteins to the plasma membrane to activate signalling pathways leading to the EMT pathway, as well as promoting actin polymerisation, which facilitates cell invasion. Thus, SH2 is critical for both suppressing E-cadherin expression and for promoting invasion. The SNAIL family of transcription factors are known inducers of EMT and have been shown to bind to E-cadherin promoter and suppress E-cadherin expression in many epithelial cell types during EMT [4,39,40]. A549^GRB2^ cells were found to express SNAIL, both at the mRNA and protein level, even before TGF-β1 stimulation, suggesting that overexpression of GRB2 upregulates the expression of SNAIL and may be responsible for the reduced E-cadherin expression observed in GRB2 overexpressing cells.

Our in vitro data suggested that GRB2 enhanced A549 cell invasion and migration along with EMT, implying a pro-oncogenic role for GRB2, especially at the later stages of metastasis. An in vivo metastasis assay was employed to reiterate our in vitro results by injecting A549^GRB2^ cancer cells into the tail vein of immunocompromised mice leading, primarily, to the formation of lung metastases. Taking into account that A549 is a metastatic cell line, the control group also formed metastatic nodules or lesions primarily in the lung. However, our in vivo metastasis model system showed a significant increase in the number of lung nodules or lesions on the surface of lungs, as well as in the number of lung metastases in lung tissue sections in A549^GRB2^-injected mice, compared to the control group. The experimental metastases model strengthens our hypothesis that GRB2 plays a late-stage tumour-promoting role both in vitro and in vivo. Its exact mechanism of action still needs to be explored.

In summary, we have shown, for the first time, that overexpression of GRB2 reduces E-cadherin expression, one of the hallmarks of EMT, in A549 lung cancer cells. We have also shown that overexpression of GRB2 promotes in vivo lung metastasis. We have shown that the N-terminal SH3 regulates E-cadherin expression probably by regulating the expression of SNAIL and the C-terminal SH3 domain regulates invasion, probably by regulating the actin cytoskeleton. We found that the central SH2 domain regulates both E-cadherin expression and invasion probably by regulating the localisation of GRB2 protein complexes through its interaction with phosphorylated tyrosine residues. It is not clear yet whether GRB2 can directly interact with SNAIL or independently activate signalling pathways in the cells that can increase SNAIL expression, and this will be the focus of future studies.

## Supplementary Materials

The following are available online at http://www.mdpi.com/2073-4409/7/8/97/s1, Figure S1: TGF-β1 treatment did not enhance transcription of GRB2 mRNA. Figure S2: Knockdown of GRB2 did not cause any change to TGF-β1 induced EMT in A549 cells. Figure S3: GRB2 overexpression causes activation of ERK, on TGF-β1 stimulation, in A549 cells.
