# Supplementary material for: Overexpression of GRB2 Enhances Epithelial to Mesenchymal Transition of A549 Cells by Upregulating SNAIL Expression
Source: Cells. 2018 Aug 7;7(8):97. doi: 10.3390/cells7080097 (PMC6116178; doi:10.3390/cells7080097)
Supplement: Supplementary file 1 [file cells-07-00097-s001.pdf]

Fig S1

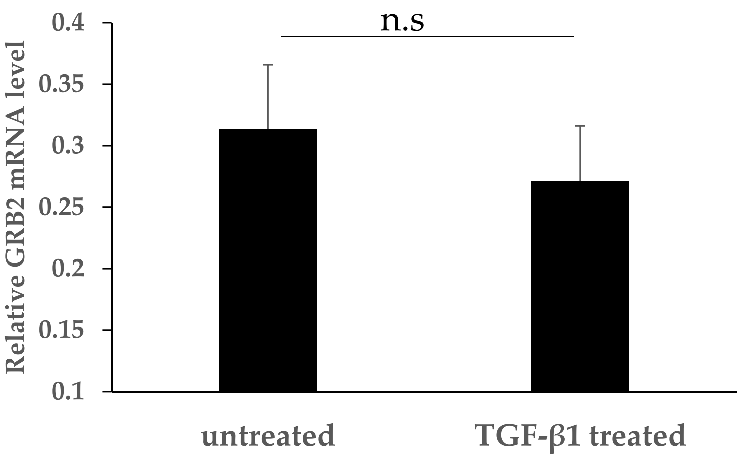

Fig 1. TGF-β1 treatment did not enhance transcription of GRB2 mRNA. Total RNA was isolated from untreated and TGF-β1 treated (5 ng/ml) A549 cells, converted to cDNA and RT-PCR was carried out using human GRB2 specific primers and human MRPL27 (control).

Fig S2

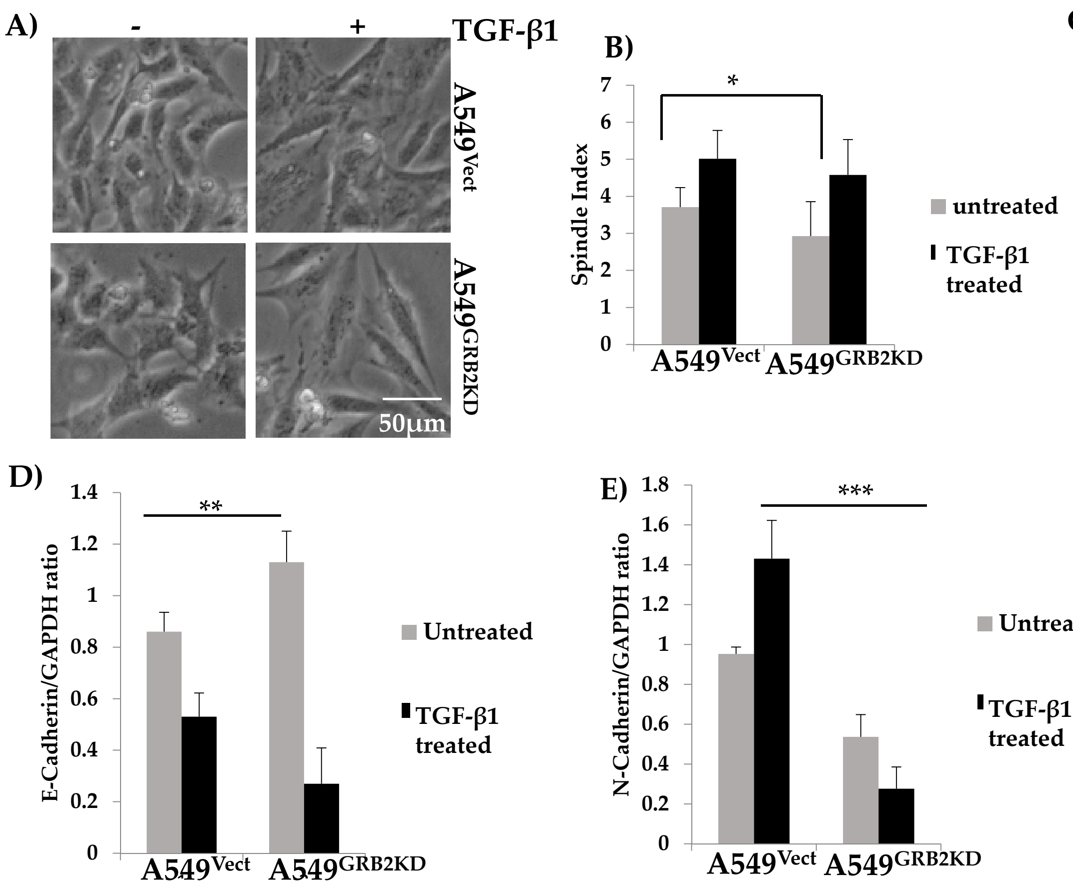

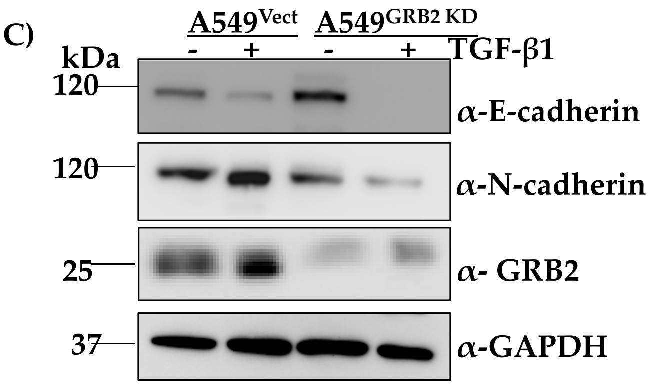

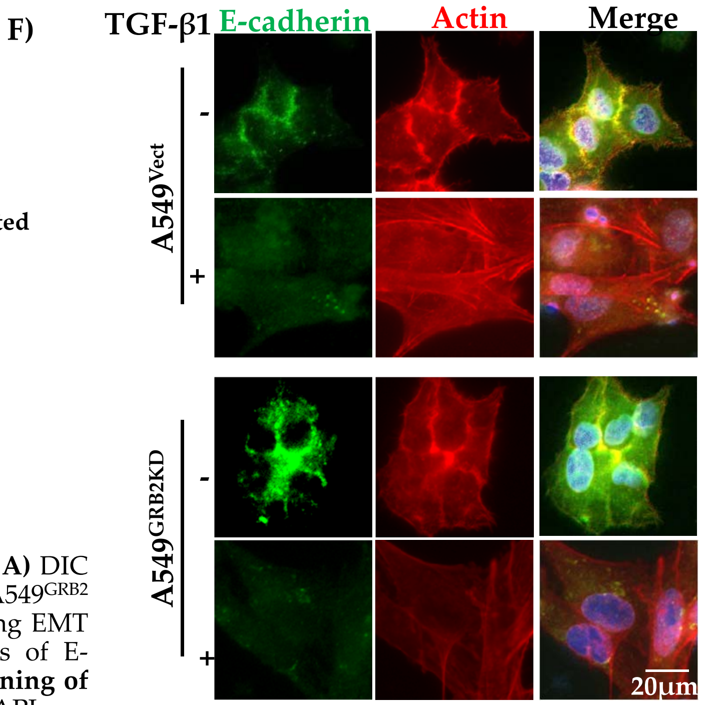

Figure 2. Knockdown of GRB2 did not cause any change to TGF-β1 induced EMT in A549 cells. A) DIC images of A549^{Vect} andA549$^{GRB2\ KD}$ cells after TGF-β1 induced EMT; **B)** Spindle Index of A549^{Vect} and A549$^{GRB2\ KD}$ cells during TGF-β1 stimulation; **C)** Western blot analysis of A549^{Vect} and A549$^{GRB2\ KD}$ cells during EMT using antibodies against E-cadherin, N-cadherin, GRB2 and GAPDH; **D)** Densitometric analysis of E-cadherin expression level; **E)** N-cadherin expression level from panel C (***p<0.001); **F) Immunostaining of** A549^{Vect} and A549$^{GRB2\ KD}$ during TGF-β1 stimulation using antibodies against E-cadherin ,actin and DAPI.

Fig S3

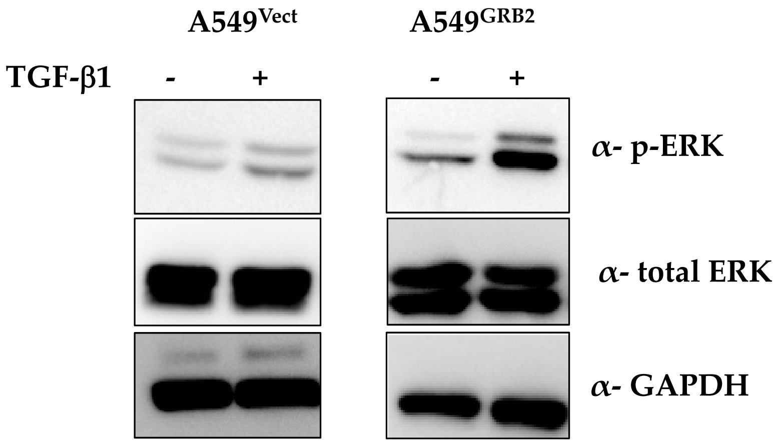

Fig 43 GRB2 overexpression causes activation of ERK, on TGF-β stimulation, in A549 cells. A549^{Vect} and A549^{GRB2} cells were seeded and then serum starved for 12 hrs. After that, vehicle (DMSO), and TGF-β1+ vehicle, were added to the cells accordingly. After 2 days, cells were lysed and used for Western blot analysis to probe for pERK and total ERK.
